# Modeling post-transcriptional regulation activity of small non-coding RNAs in *Escherichia coli*

**DOI:** 10.1186/1471-2105-10-S4-S6

**Published:** 2009-04-29

**Authors:** Rui-Sheng Wang, Guangxu Jin, Xiang-Sun Zhang, Luonan Chen

**Affiliations:** 1 School of Information, Renmin University of China, Beijing 100872, PR China; 2 Academy of Mathematics and Systems Science, CAS, Beijing 100190, PR China; 3 Institute of Systems Biology, Shanghai University, Shanghai 200444, PR China; 4 Osaka Sangyo University, Osaka 574-8530, Japan

## Abstract

**Background:**

Transcriptional regulation is a fundamental process in biological systems, where transcription factors (TFs) have been revealed to play crucial roles. In recent years, in addition to TFs, an increasing number of non-coding RNAs (ncRNAs) have been shown to mediate post-transcriptional processes and regulate many critical pathways in both prokaryotes and eukaryotes. On the other hand, with more and more high-throughput biological data becoming available, it is possible and imperative to quantitatively study gene regulation in a systematic and detailed manner.

**Results:**

Most existing studies for inferring transcriptional regulatory interactions and the activity of TFs ignore the possible post-transcriptional effects of ncRNAs. In this work, we propose a novel framework to infer the activity of regulators including both TFs and ncRNAs by exploring the expression profiles of target genes and (post)transcriptional regulatory relationships. We model the integrated regulatory system by a set of biochemical reactions which lead to a log-bilinear problem. The inference process is achieved by an iterative algorithm, in which two linear programming models are efficiently solved. In contrast to available related studies, the effects of ncRNAs on transcription process are considered in this work, and thus more reasonable and accurate reconstruction can be expected. In addition, the approach is suitable for large-scale problems from the viewpoint of computation. Experiments on two synthesized data sets and a model system of *Escherichia coli *(*E. coli*) carbon source transition from glucose to acetate illustrate the effectiveness of our model and algorithm.

**Conclusion:**

Our results show that incorporating the post-transcriptional regulation of ncRNAs into system model can mine the hidden effects from the regulation activity of TFs in transcription processes and thus can uncover the biological mechanisms in gene regulation in a more accurate manner. The software for the algorithm in this paper is available upon request.

## Background

Transcription regulation of gene expression is one of the most important processes in molecular biology. It transmits static information encoded in the DNA sequence into functional protein molecules which in turn control most of the cellular processes. It is some DNA-binding proteins known as transcription factors (TFs) that achieve the transcriptional regulation of genes. TFs usually attach to specific DNA promoter regions to exert their effects positively or negatively on binding of RNA polymerase to the promoter region of a gene. The process of gene expression involves a series of complex biochemical events such as transcription, cooperativity and competition of multiple TFs, intron splicing, translation, post-translational modification, degradation and other mechanisms. So far, there have been great efforts contributed to identify transcription factors and generate binding data for many organisms [1,2]. Another equally important problem is to synthesize and analyze transcriptional regulatory networks from ChIP-chip data and gene expression profiles [3-5]. More detailed surveys about these topics can be found in [6,7].

Generally, the ability of a TF in regulating a target gene is determined by its activity, i.e. the active concentration after various post-translational modifications. Understanding the activity of TFs is fundamental to elucidate the underlying mechanism in transcription regulation. However, although many routine techniques are available to measure the expression profiles of thousands of genes simultaneously, there is currently no a reliable experiment technology to routinely measure the activities of regulators due to the complexity of post-translational process. The expression of a gene encoding a TF provides only limited information about activity, since various post-translational modifications heavily affect the protein concentration [8]. On the other hand, since the expression profiles of target genes represent the regulation results of their regulators, a lot of computational works have been made to infer TF activity from their target gene expression profiles and TF-gene regulatory relationships. Liao et al. and Kao et al. made the first attempt to infer regulator activities by combining gene expression data of target genes and ChIP-chip data [9,10]. They developed a matrix decomposition method called network component analysis (NCA) to determine transcription regulator activity. This method was further extended as partial least squares (PLS) based network component analysis by Boulesteix and Strimmer [11] which offers an efficient and sound way to infer regulator activity for any given connectivity matrix without much restriction like NCA. Tran et al. derive a generalized form of NCA called gNCA which expands the capability of transcriptional network analysis by incorporating regulatory signal constraints arising from genetic knockouts [12]. Based on a same system model, a mixed integer linear programming approach is developed to infer transcription factor activity in [13] which can easily integrate prior knowledge about regulatory relationships. In addition, Nguyen and D'haeseleer [14] developed a matrix factorization method to decompose gene expression matrix which can obtain motif strength and TF activity profiles simultaneously. Pournara and Wernisch [15] studied five factor analysis methods for predicting protein activities of TFs. Other related work can be found in [6,16].

In addition to coding genes and TFs, in recent years, the biological roles of non-coding RNAs (ncRNAs) that are transcribed from DNA but not translated into proteins have been widely studied [17,18]. Especially, small non-coding RNAs (sRNAs) have been demonstrated to play critical roles in regulating gene expression [19]. MicroRNA (miRNA), a family of sRNAs with a single-stranded RNA molecule of about 18–24 nucleotides in length, was initially discovered as small temporal RNAs that regulate developmental transitions in *C. elegans*, and now found to have diverse expression patterns and probably regulate many aspects of development and physiology [18]. miRNAs are predicted to regulate the expression of approximately one-third of all human genes and play important roles in coordinating many cellular processes, particularly those involved in development and disease including various cancers, acting either as oncogenes or tumor suppressor genes [20-22]. Many computational methods available for predicting the mRNA targets of miRNAs indicate that an miRNA could target tens to hundreds of genes [23,24]. Although the detailed regulation mechanisms of sRNAs are largely unknown, some of them already have characterized targets and have been recognized to negatively regulate the expression of target genes at the post-transcriptional level by base pairing with mRNAs through binding to mRNA targets, leading to target degradation or inhibition of translation [19,25-27].

With an increasing number of ncRNAs being shown to mediate post-transcriptional processes and regulate critical pathways in prokaryotes and eukaryotes, quantitatively characterizing their regulation roles in gene expression is a new and important task [28-30]. For example, Shimoni et al. used dynamical simulations to characterize the regulation modes of sRNAs and compared them with the transcriptional regulation mediated by TFs and post-translational regulation achieved by protein interactions [28]. Levine et al. adopted a quantitative approach to study bacterial sRNAs in *E. coli *and found that the mode of gene regulation of sRNAs is distinct from that of TF regulation [29]. Mehta et al. quantitatively compared sRNAs with conventional TFs by calculating the steady-state behavior, noise properties, amplification, and dynamical response to large input signals of both forms of regulation [30]. Aguda et al. studied a feedback loop involving a miRNA cluster and two TFs and showed the oncogenic and tumor suppressor properties of miR-17–92 [31]. Khanin et al. developed a kinetic model of post-transcriptional regulation of miRNAs and focused on studying the miRNAs' effect on mRNAs degradation rates by inferring kinetic parameters using a temporal microarray dataset [32]. Although there are many efforts for exploring the regulation properties of individual miRNAs and comparing them with TF regulation from a dynamic view, few work is developed on integrating the post-transcriptional regulation of sRNAs into TF regulation and creating a comprehensive regulatory network to investigate gene regulation in an overall manner.

In light of existing work for studying transcriptional regulation and regulator activities that ignores the possible post-transcriptional effects of sRNAs on mRNA level, in this paper, we propose a novel approach to infer the activity of regulators including TFs and sRNAs. The new framework explores target gene expression profiles and integrated two-level (transcription and post-transcription) regulatory relationships, and thus can incorporate the regulatory effects of sRNAs into the inference process, making the reconstructed network more biologically reasonable and meaningful. We model the integrated regulatory system by a set of biochemical reactions which lead to a log-bilinear problem. Then an iterative algorithm is developed to address the system model, in which two linear programming (LP) problems are effectively solved, making the framework suitable for large-scale instances. Since the regulatory role of sRNAs in bacteria has actually been a subject of active research for the last several decades, we test our model and algorithm by using *E. coli *data and available information from previous research studies. Experiments on two synthesized data sets and a real data set about a model system of *E. coli *carbon source transition from glucose to acetate illustrate the effectiveness of our model and algorithm.

## Results

**Figure 1:**
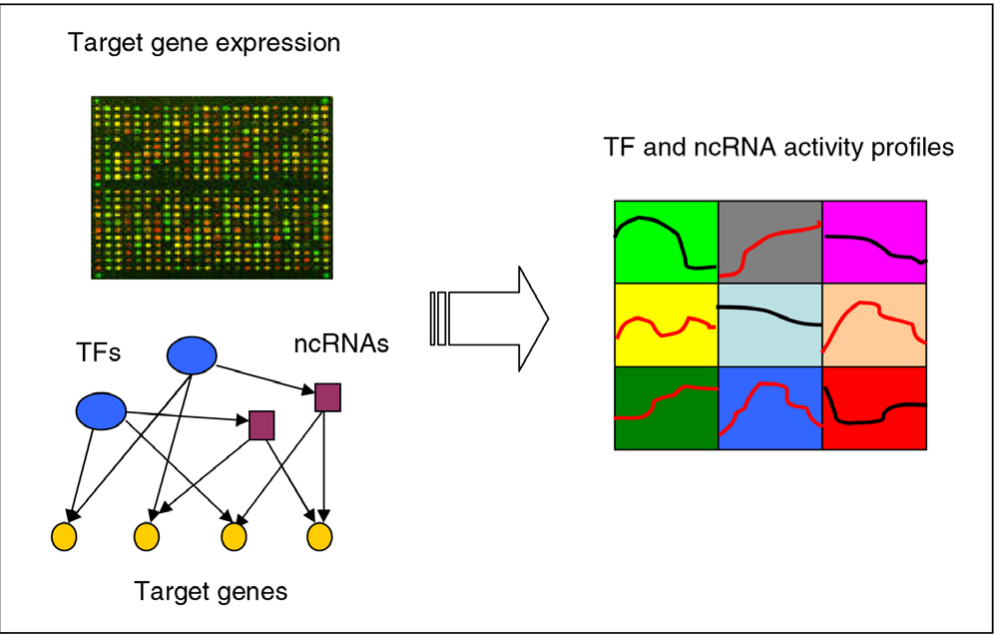
**Scheme of inferring post-transcription regulation activity**.

As mentioned in the last section, the activity of regulators (the active concentration of regulators) determines their ability in regulation of target genes. On the other hand, the expression profiles of target genes represent the regulation results of regulators. Therefore, the regulator activities can be retrieved from the expression profiles of their target genes and the corresponding regulatory relationships. In this work, we collect the regulatory interactions between TFs, ncRNAs and target genes and aim to infer the concentrations of TF and ncRNAs from the mRNA levels of target genes and regulatory network structure. Figure 1 illustrates the main step of the procedure. Clearly from the biological viewpoint, it is reasonable and biologically meaningful to incorporate the regulation effects of post-transcription on mRNAs when inferring regulator activities since many ncRNAs are found to downregulate target genes.

Quantitative reconstruction of regulatory activities needs a biologically meaningful mathematical model to describe the relationships between the activities of regulators (especially ncRNAs here), target gene expression levels, and regulatory network structure. Since transcription and post-transcription are achieved by a series of biochemical reactions with TFs, ncRNAs, mRNAs and proteins as reactants, we can construct a model from the set of involved biochemical reactions. Then, based on different kinetics such as Michaelis-Menten kinetics and mass action kinetics, we can obtain mathematical models at different levels. In this paper, we adopt the widely used mass action kinetics to mathematically formulate the integrated regulatory system.

### Integrated system model

Transcriptional regulation and post-transcriptional regulation on gene expression can be modeled as a closed reacting system, in which proteins, DNA, mRNAs, ncRNAs and other intermediate species are components of the biochemical system. In transcription process, independent TFs or interacting TFs bind to DNA sequences so as to recruit RNA polymerase II (RPII) onto promoter region of DNA through a set of reversible reactions. Although the species involving in transcription regulation may also take part in other independent reactions, these reactions are usually much faster compared with those in transcription [4]. We can assume that they reach equilibrium, i.e. the amounts of atomic species are conserved in this closed system. Therefore, an overall chemical reaction of transcription initiation can be given by

(1) $${\text{DNA}}_{i}+J_{i1}\cdot {\text{TF}}_{1}+\cdots +J_{ic}\cdot {\text{TF}}_{c}+\text{RPII}\overset{k_{1}}{\underset{k_{-1}}{⇌}}{\text{DNA}}_{i}{\left({\text{TF}}_{1}\right)}_{J_{i1}}\cdots {\left({\text{TF}}_{c}\right)}_{J_{ic}}\text{RPII},$$

where there are totally *c *TFs regulating gene *i*, the stoichiometric coefficient *J*_*ij*_, *j *= 1, 2, ⋯, *c *represents the effective abundance of TF_*j *_involved in the regulation of gene *i*, and DNA_*i *_is the sequence of gene *i*. *k*_1 _and *k*_-1 _are the rate constants of forward reaction and reverse reaction respectively. ${\text{DNA}}_{i}{\left({\text{TF}}_{1}\right)}_{J_{i1}}\cdots {\left({\text{TF}}_{c}\right)}_{J_{ic}}\text{RPII}$ denotes the immobilized compound formed by DNA, TFs and RNA polymerase II. After transcription initiation, mRNAs of gene *i *are synthesized through the following irreversible reaction

(2) $${\text{DNA}}_{i}{\left({\text{TF}}_{1}\right)}_{J_{i1}}\cdots {\left({\text{TF}}_{c}\right)}_{J_{ic}}\text{RPII}\overset{k_{2}}{\to }{\text{mRNA}}_{i}.$$

where *k*_2 _is the rate constant of the reaction.

If no post-transcriptional events exert effects on the degradation of mRNAs or the inhibition of translation, or if we do not consider the effects of post-transcriptional events, we can directly establish a mathematical model describing the concentration changes of mRNAs according to above reactions. Now, we stress the regulatory roles of ncRNAs in post-transcriptional process. As existing literature stated, many ncRNAs have characterized targets and negatively regulate mRNAs by binding to the target mRNAs and destabilizing them in a process mediated by the RNA chaperone Hfq (Sm-like host factor I) [29]. After binding, both sRNAs and mRNAs are degraded by pairing Hfq at a rate that depends on the sRNA-mRNA regulation strength [19,33], Therefore, we model the regulation effects of ncRNAs on mRNAs in the post-transcription process by the following biochemical reaction

(3) $$M_{i1}\cdot {\text{ncRNA}}_{1}+\cdots +M_{ik}\cdot {\text{ncRNA}}_{k}+{\text{mRNA}}_{i}\overset{k_{3}}{\to }{\left({\text{ncRNA}}_{1}\right)}_{M_{i1}}\cdots {\left({\text{ncRNA}}_{k}\right)}_{M_{ik}}{\text{mRNA}}_{i}$$

where *M*_*is*_, *s *= 1, 2, ⋯, *k *in the above reaction is the stoichiometric coefficient and *k*_3 _is the rate constant of the reaction. Though the formation of sRNA-mRNA complex is irreversible and may be noncatalytic, we use the above equation to represent the regulation effects of ncRNAs which are viewed as a kind of degradation of mRNAs.

Mass action law means that the rate of any given elementary reaction is proportional to the product of the concentrations of the reactants. According to mass action law, the concentration changes of mRNAs and ${\text{DNA}}_{i}{\left({\text{TF}}_{1}\right)}_{J_{i1}}\cdots {\left({\text{TF}}_{c}\right)}_{J_{ic}}\text{RPII}$ can be described as the following equations

(4) $$\frac{d[{\text{mRNA}}_{i}]}{dt}=k_{2}[{\text{DNA}}_{i}{\left({\text{TF}}_{1}\right)}_{J_{i1}}\cdots {\left({\text{TF}}_{c}\right)}_{J_{ic}}\text{RPII}]-k_{3}\prod_{s=1}^{k}{\left[{\text{ncRNA}}_{s}\right]}^{M_{is}}[{\text{mRNA}}_{i}],$$

(5) $$\frac{d[{\text{DNA}}_{i}{\left({\text{TF}}_{1}\right)}_{J_{i1}}\cdots {\left({\text{TF}}_{c}\right)}_{J_{ic}}\text{RPII}]}{dt}={k^{\prime }}_{1}\prod_{j=1}^{c}{\left[{\text{TF}}_{j}\right]}^{J_{ij}}-k_{-1}[{\text{DNA}}_{i}{\left({\text{TF}}_{1}\right)}_{J_{i1}}\cdots {\left({\text{TF}}_{c}\right)}_{J_{ic}}\text{RPII}].$$

where [·] represents the concentration of the corresponding species, and ${k^{\prime }}_{1}$ = *k*_1 _[DNA_*i*_] [RPII]. In the second term of equation (4), ${\left[{\text{ncRNA}}_{s}\right]}^{M_{is}}$ is exactly like the degradation factor in the regulation model used in [12], in which degradation factors are discarded. By assuming that the closed reaction system attains equilibrium (or considering a time scale in which quasi-steady state approximation is valid) and that there are sufficient RPII in cells so that [RPII] = 1 (i.e. the normalized concentration) and [DNA_*i*_] remains constant, we have the following equation according to the equilibrium form of (4)–(5)

$$[{\text{mRNA}}_{i}]\propto \prod_{j=1}^{c}{\left[{\text{TF}}_{j}\right]}^{J_{ij}}\cdot \prod_{s=1}^{k}{\left[{\text{ncRNA}}_{s}\right]}^{-M_{is}}.$$

After introducing the status of *t *= 0 as a reference sample, we obtain the following log-bilinear model

$$\frac{x_{i}(t)}{x_{i}(0)}={\prod_{j=1}^{c}\left(\frac{A_{j}(t)}{A_{j}(0)}\right)}^{J_{ij}}\cdot \prod_{s=1}^{k}{\left(\frac{R_{s}(t)}{R_{s}(0)}\right)}^{-M_{is}},$$

where *x*_*i*_(*t*) = [mRNA_*i*_](*t*), *A*_*j*_(*t*) = [TF_*j*_](*t*), *R*_*s*_(*t*) = [ncRNA_*s*_](*t*). It can be formulated as the following bilinear model in a matrix form through log transformation

(6) $$\begin{array}{c} X_{m\times n}=J_{m\times c}A_{c\times n}-M_{m\times k}R_{k\times n}\\ =\left[\begin{array}{cc} J & -M \end{array}\right]\left[\begin{array}{c} A\\ R \end{array}\right], \end{array}$$

where *X*_*m *× *n *_is an *m *× *n *matrix with element log(*x*_*i*_(*t*)/*x*_*i*_(0)) for *i *= 1, ⋯, *m*, *t *= 1, ⋯, *n*; *J *is an *m *× *c *matrix with element *J*_*ij *_for *i *= 1, ⋯, *m*, *j *= 1, ⋯, *c*; *M *is an *m *× *k *matrix with element *M*_*is *_for *i *= 1, ⋯, *m*, *s *= 1, ⋯, *k*; *A *is a *c *× *n *matrix with element log(*A*_*j*_(*t*)/*A*_*j*_(0)) for *j *= 1, ⋯, *c*, *t *= 1, ⋯, *n*; *R *is a *k *× *n *matrix with element log(*R*_*s*_(*t*)/*R*_*s*_(0)) for *s *= 1, ⋯, *k*, *t *= 1, ⋯, *n*. Generally, most non-zero entries of *M *are positive because ncRNAs usually negatively regulate the expression of mRNAs. Equation (6) is a model with *m *genes (mRNAs), *k *ncRNAs, *c *TFs, and their concentrations with *n *time points.

In this model, [*J *-*M*] represents a two-level regulatory network involving both transcription (mediated by TFs) and post-transcription (mediated by ncRNAs), with each row corresponding to a target gene and each column corresponding to a regulator. In this work, the two-level regulatory network is partially known, i.e. the topological structure can be accessed from databases, but the numerical regulation strength is to be inferred by the model. Our goal is mainly to reconstruct the activities of regulators *A *and *R *from the expression profiles of target genes *X*. The reconstruction process is formulated into an optimization problem and solved by a proposed iterative algorithm (see Methods).

### Illustration of the model by a hypothetical network

**Figure 2:**
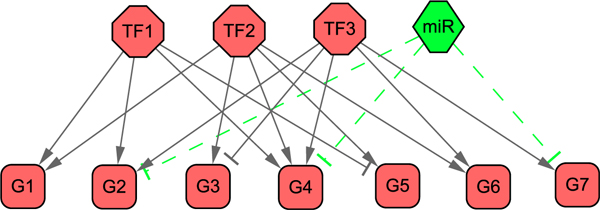
**A simple transcriptional regulatory network with three TFs, one miRNA and seven target genes**.

**Figure 3:**
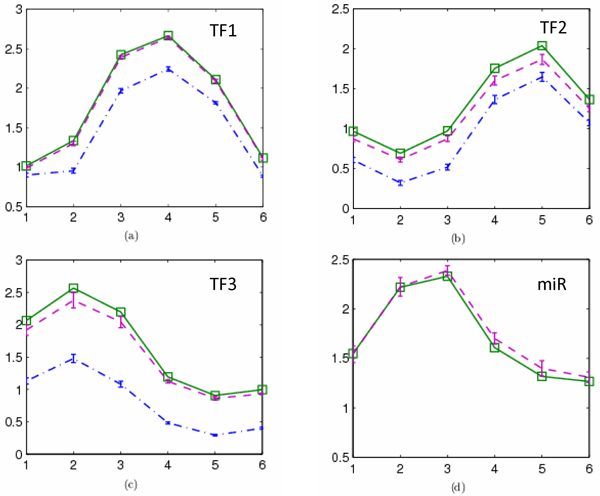
**Comparison of the inferred regulatory activities with/without incorporating the regulation of miRNA with the true values**. The squares with solid line are the true values. The line -. with error bars corresponds to the reconstructed regulator activities without considering miRNA. The dashed line with error bars corresponds to the reconstructed regulator activities without considering miRNA.

We first use a hypothetical network to illustrate our model and motivation of incorporating sRNAs. The simple network is given in Figure 2, which consists of three TFs (*c *= 3), and one miRNA (*k *= 1) regulating seven genes (*m *= 7). From a set of preassigned regulation strengths of regulators and their regulation activities with six time samples (*n *= 6), the expression profiles of target genes (the matrix *X*) are generated numerically with a Gaussian white noise *N*(0,0.05) that simulates experimental microarray gene expression data. With the synthesized expression profiles of target genes and regulatory network structure, we reconstruct the regulator activities (the matrices *A *and *R*). The synthesized data can be found in Additional file 1. To mimic the fact that ChIP-chip data can only provide rough regulation strength by giving *p*-values of TF-gene bindings, we use the original regulation strengths with a large random noise of uniform distribution (15%) to construct an initial regulation matrix for the matrices *J *and *M*. To illustrate the effects of the miRNA on reconstruction accuracy, we first assume that only three TFs are known to regulate the genes without the knowledge of the post-transcriptional regulation effects of the miRNA. And then, we examine the case that considers the regulation of miRNA. After constructing the system model (6), we use the iterative algorithm to solve the model (Methods). The parameter *λ *in this small example is simply set as 1. Since the iterative algorithm starts from random initial matrices, we rerun the algorithm for five times, and both mean values and standard variation of the reconstruction results are summarized in Figure 3. We can see that although we add noises into target expression profiles and use largely perturbed regulation matrices as initial solutions, the reconstructed regulator activities have a good agreement with the true values. However, if we ignore the regulation effects of miRNA, the inference accuracies are heavily weakened. An observable consequence is that the TF activities are underestimated if miRNA regulation is ignored, which can be confirmed in the following real data in *E. coli*. Here the simple network only contains a single miRNA. In real networks, if many ncRNAs have post-transcriptional regulation effects on target genes, not only the amplitudes of reconstructed TF activities but also the whole dynamics will be changed without incorporating post-transcriptional events.

### Reconstruction of absorbance spectra of hemoglobin solutions

In this section, we use a network of seven hemoglobin solutions (denoted by *M*_1_, *M*_2 _⋯, *M*_7_) and their absorbance spectra which were measured in Liao et al. [9] to evaluate our method. This data set has been widely used to test matrix factorization methods [12,15]. Each of these seven solutions contains a combination of three components: oxyhemoglobin, methemoglobin and cyano-methemoglobin. The absorbance spectra were taken between 380 and 700 nm with 1-nm increments. According to Beer-Lambert law, the absorbance spectra of the mixture can be described as a linear combination of the composition proportions of three components and the absorbance spectra of each pure solution according to a certain mixing diagram [9]. The mixing diagram represents the compositions of pure components, which serves as the regulatory network. The absorbance spectra of seven mixed hemoglobin solutions serve as the expression profiles of targets, and the three pure components serve as regulators. Now we test if or not our iteration algorithm can correctly infer the absorbance spectra of each pure solution (serving as the activities of regulators) by using those of mixed solutions and their mixing diagram.

**Figure 4:**
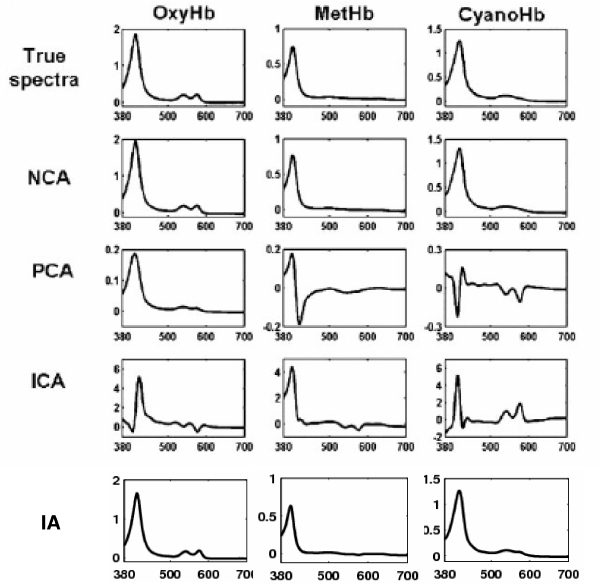
**Validation of our method using absorbance spectra of hemoglobin solutions**. where OxyHb, oxyhemoglobin; MetHb, methemoglobin; CyanoHb, cyano-methemoglobin; IA: our iterative algorithm.

Since the iteration algorithm starts from random initial matrices, the convergence results may be different upon different implementations. We solve this problem by rerunning the algorithm for certain times and then averaging the results. To evaluate the performance of the method, we compared it with those from Network Component Analysis (NCA), Principle Component analysis (PCA), Independent Component Analysis (ICA). The comparison results on this dataset are summarized in Figure 4, where IA denotes our iteration algorithm. Clearly, the results in Figure 4 show that both our algorithm and NCA can well retrieve the regulatory signals (pure component spectra) since they agree well with the true spectra obtained from independent measurements of pure components. In contrast, PCA or ICA cannot reconstruct the pure component spectra with a good accuracy. The results confirm the effectiveness of our iteration algorithm. Compared with NCA, the peak regions of the spectra for oxyhemoglobin and methemoglobin solutions reconstructed by our method are slight lower. However, our algorithm has no any restrictions on data matrix *X *and factorized matrices *J*, *A*. In contrast, there are several restriction conditions to make NCA feasible [9]. If these conditions are not satisfied, the connection matrix *J *must be reduced, which restricts the ability of NCA in applying to arbitrary datasets in practice.

### Inference of regulator activities in *E. coli *carbon source transition

**Figure 5:**
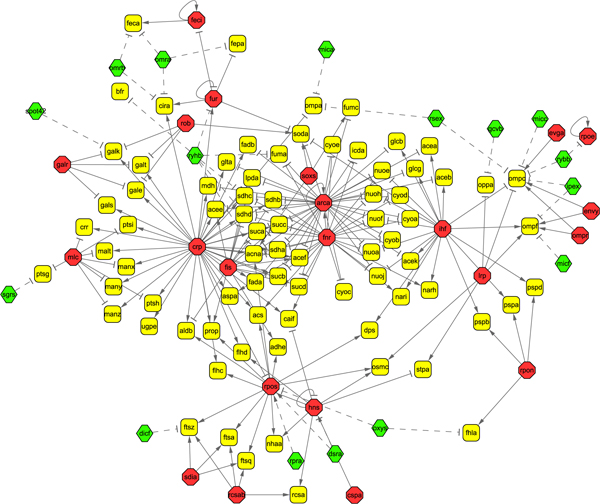
**The assembled two-level regulatory networks during glucose to acetate transition in *E. coli***. The solid lines denote transcriptional regulation, and the dashed lines represent post-transcriptional regulation. The octagons denote transcription factors, the hexagons represent ncRNAs and the rectangles are target genes. The sharp arrows denote activation and the blunt arrows denote inhibition.

Finally, we applied our model and method to infer the regulator activities in *E. coli *carbon source transition from glucose to acetate. We first assemble a two-level network including both transcriptional regulation and post-transcriptional regulation from available data sources. RegulonDB is a database storing the transcription information of *E. coli *K12 [34]. In this database, there are 160 transcription factors and 3154 TF-gene interactions (transcriptional regulatory relationships). The ncRNA-protein interaction database (NPInter) is a database storing ncRNA-protein interactions which cover eight category functional interactions in six model organisms [35], among which 'the ncRNA regulates the mRNA' and 'the ncRNA is regulated by the protein' are interactions involving in transcriptional process and post-transcriptional process. TF-gene interactions and ncRNA-mRNA interactions can be combined into a two-level regulatory network with common targets as connectors. There are 47 ncRNA-mRNA interactions and 22 regulator-ncRNA interactions for *E. coli *in NPInter. These numbers are much larger than those from other five organisms. The ncRNA-mRNA interactions in [28] that are not covered by NPInter are also incorporated into our research. We use the gene expression data of *E. coli *carbon source transition from glucose to acetate [10] which have 10 time points to infer the activities of the regulators (TFs and ncRNAs) in this biological process. Among the genes involving in *E. coli *transcriptional regulatory networks, 296 of them were shown to be perturbed during transition from glucose to acetate growth [10]. According to the collected ncRNAs, TFs and theirs targets, we further reduce the targets as a set of 150 genes. Finally, a test data set with 38 regulators (22 TFs and 16 ncRNAs) and 150 target genes is collected. The assembled two-level regulatory network is illustrated in Figure 5, where the target genes that are regulated by a single TF are not shown due to the largeness of the network. The whole two-level regulatory network can be found in Additional file 2. The regulatory interactions that we collected are from manually curated databases [34,35]. They are observed in biological experiments and have high confidences, so we do not need to make the assembled two-level regulatory network sparser. Therefore, here we just set *λ *as 0. If predicted regulatory interactions are used (e.g. predicted miRNA targets), we use *λ *to control the sparseness of network structure. Since no routine biological techniques are available for measuring regulator activities, there is no gold standard to evaluate the inferred results. Instead, we conducted biological analysis by comparing the results based solely on transcriptional events in [10] and [12]. Such an evaluation scheme is effective because identical experimental gene expression data and transcriptional regulatory network are used. The only difference is that we additionally consider the regulation effects of sRNAs.

**Figure 6:**
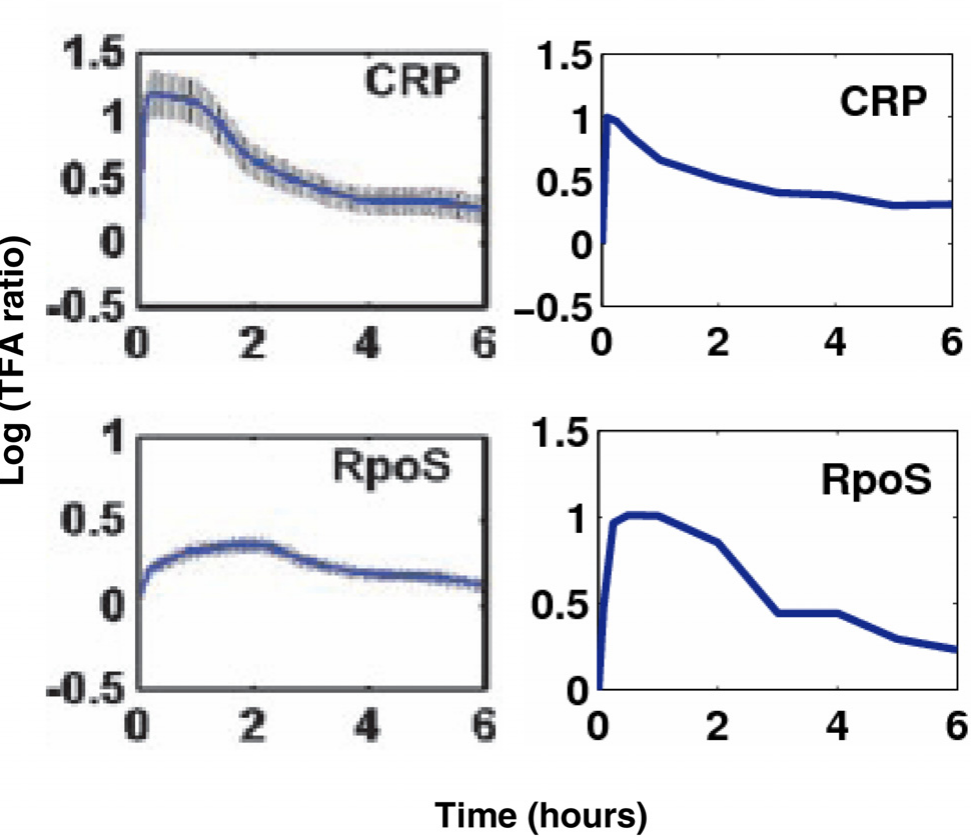
**The activity dynamics of CRP and Rpos during glucose to acetate transition in *E. coli***. Left: without considering post-transcriptional events; Right: with considering post-transcriptional events.

Figure 6 lists the reconstructed activity dynamics of two transcription factors CRP and Rpos during glucose to acetate transition, along with those inferred by considering only transcriptional events. CRP is an *E.coli *transcription factor which has 64 target genes involving in the carbon source transition. It requires the binding of the signal metabolite cAMP for activation [36]. The transcription activity profile of CRP actually represents that of the CRP-cAMP complex which obviously cannot be approximated by the gene expression profile of CRP. We retrieved the activity of CRP by the expression profiles of its target genes. From Figure 6, we can see that CRP has very similar dynamics under two situations. This is mainly because CRP has too many target genes, only one of its targets is also regulated by sRNAs. Therefore, the effect of post-transcriptional events is not significant. As another example, RpoS is a TF with 13 target genes involving in the carbon source transition, where 2 of them are also regulated by sRNAs. From Figure 6, we can see that the activity dynamics of RpoS are different at two situations. Its activity quantity under consideration of the effects of sRNAs is greater than original activity. This is mainly because the negative regulation effect of sRNAs is hidden into that of TFs if we only consider transcriptional events with the post-transcriptional effect ignored. Another reason is that RpoS is positively regulated by two sRNAs DsrA and RprA. Since we consider their regulation effects in our model, the activity of RpoS is naturally higher than originally reconstructed one.

**Figure 7:**
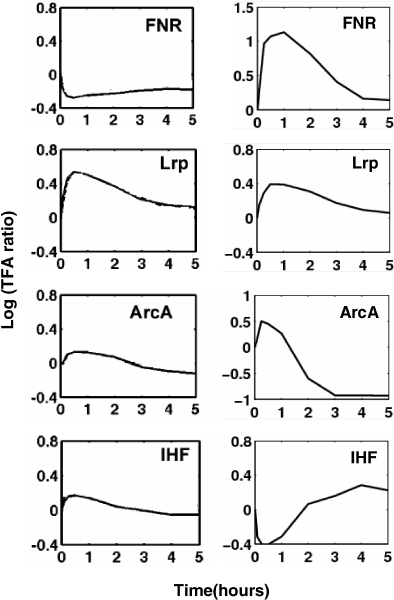
**Comparison of the activity dynamics of some TFs during glucose to acetate transition in *E. coli***. Left: without considering post-transcriptional events; Right: with considering post-transcriptional events.

**Figure 8:**
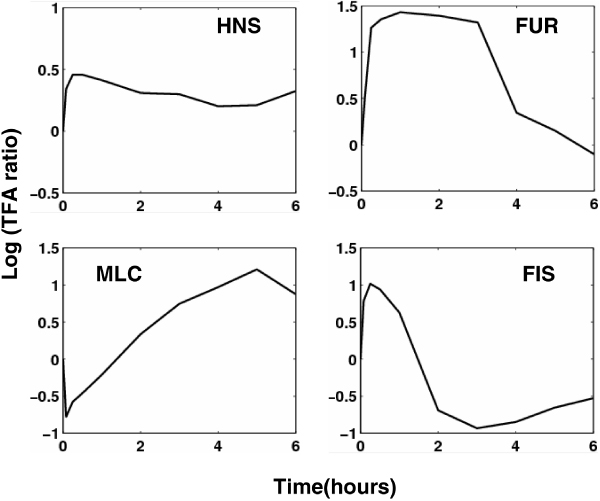
**The activity dynamics of some TFs during glucose to acetate transition in *E. coli***.

Some transcription factors in our work are not covered by [10], so we compare the reconstructed activities of these TFs with the results in [12], where for the purpose of comparison, the same time spans are used. The activity dynamics of TFs, without and with considering post-transcription, are listed in Figure 7. In *E. coli*, FHS is a major regulator controlling the physiological switch between aerobic and anaerobic growth conditions [37]. We can see that the activity dynamics of FHS is different at two situations. The activity quantity under consideration of the effects of sRNAs is much greater than original activity. Looking at the assembled two-level regulatory network, we see that FHS has at least four target genes that are also regulated by the sRNA Ryhb. Lrp is a global regulator of metabolism in *E. coli *that helps cells respond to changes in environmental conditions. In our reconstruction, the activity dynamics of Lrp under consideration of the effects of sRNAs is almost identical to the original activity. Although Lrp has several target genes that are regulated by sRNAs, these target genes have many other regulators. For example, the target gene ompc totally has 9 regulators, and ompf has 6 regulator. Therefore, the reconstructed activity of Lrp does not change much after considering post-transcription. ArcA is a global regulatory gene in *E. coli *which mediates the repression of enzymes in aerobic pathways. There is also an evidence that ArcA functions in redox regulation in *E. coli *under microaerobic but not anaerobic or aerobic conditions [38]. In our result, ArcA has similar activity dynamics under consideration or no consideration of the effects of sRNAs, i.e. within the first hour, the activity is increasing, then an hour later, the activity begins to decrease. However, the amplitudes of the activity curves are different. The reconstructed activity dynamics of IHF is slightly different at two situations within the first two hours, indicating the regulation effects of the sRNAs mainly exert in the beginning phase of glucose to acetate transition. In addition to the TFs that we analyzed above, there are some other TFs whose activities are not covered by [10] and [12]. Figure 8 lists the activity dynamic of such TFs.

**Figure 9:**
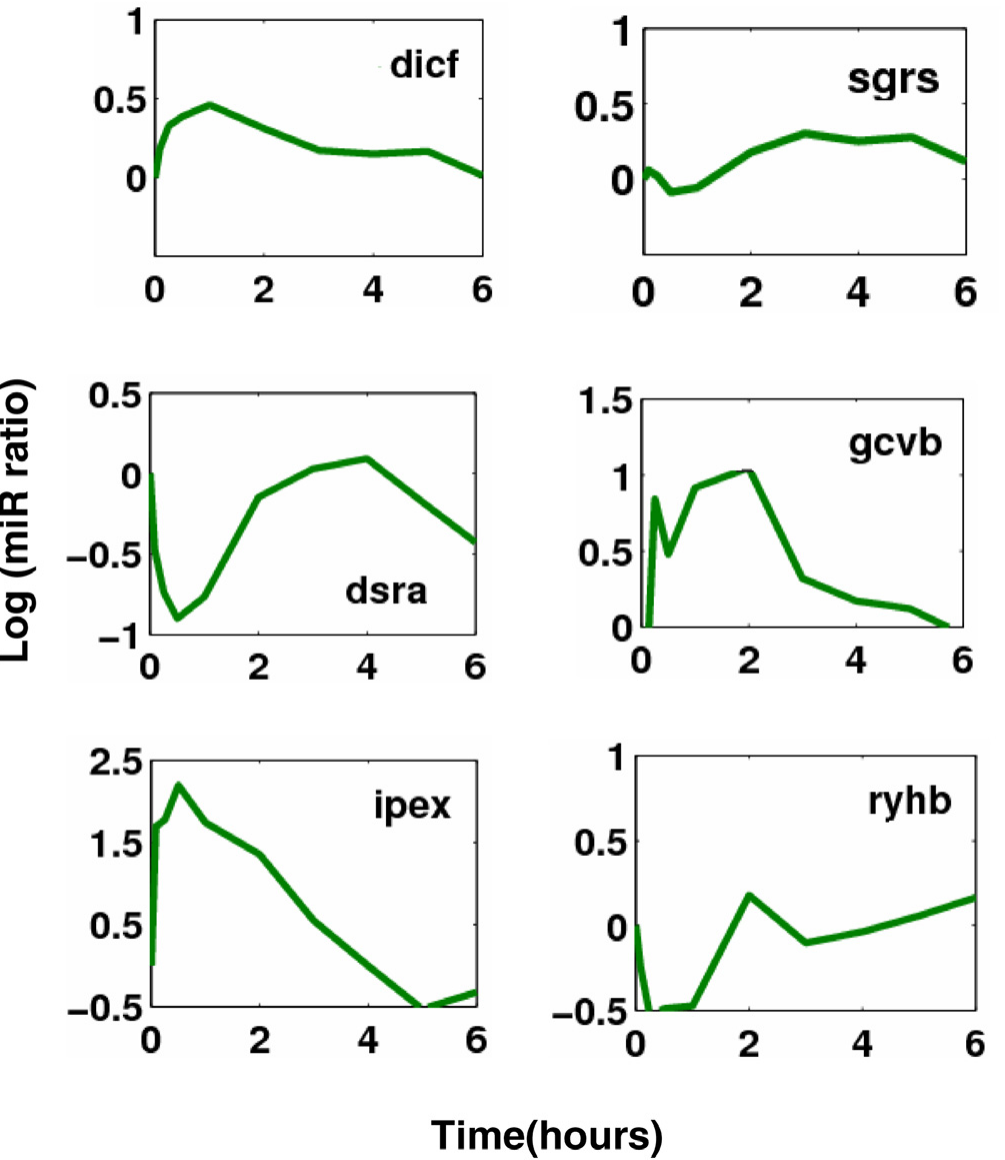
**The activity dynamics of some sRNAs during glucose to acetate transition in *E. coli***.

Aside from the activities of TFs, the post-transcriptional regulatory activities (concentrations) of ncRNAs are also reconstructed. Figure 9 illustrates the activity dynamics of some ncRNAs. dicF is an *E.coli *small RNA which blocks cell division by inhibiting ftsZ translation. Actually, dicF-like elements similar to transcriptional terminators have been found in many bacterial genomes [39]. From the reconstructed dynamics, dicF exerts an inhibition effect on its target genes in the first three hours. SgrS is a 227-nt small RNA that is expressed in *E.coli *during glucose-phosphate stress. Under stress conditions, SgrS exerts its post-transcriptional effects on glucose transporter by negatively regulating translation and stability of the ptsG mRNA (encoding the major glucose transporter) through a base pairing-dependent mechanism [40]. DsrA is an 87-nucleotide regulatory RNA of *E. coli *and has RNA-RNA interactions with two different mRNAs, hns and rpoS. DsrA has opposite effects on these transcriptional regulators, i.e. it inhibits hns and activates rpos, which leads to the fact that hns levels decrease, whereas RpoS levels increase. There are evidences that DsrA enhances hns mRNA turnover yet stabilizes rpoS mRNA [41], which is consistent with its opposite effects. RyhB is a stress-induced Hfq-binding sRNA of *E. coli*. It downregulates the expression of target mRNAs encoding Fe-binding or Fe-storage proteins through base-pairing. It has been revealed that when Fe is limiting, RyhB levels rise, and target mRNAs are rapidly degraded. RyhB turnover is coupled to and dependent on pairing with the target mRNAs [27]. Most of the other sRNAs in this study are also inhibitors and negatively regulate their targets. There are extreme few cases for sRNAs with positive regulation. DsrA and RprA are among the members of this class [19].

The reconstructed regulator activities can be used to predict the rough expression dynamics of some target genes through the model (6), provided that its regulators and their regulation nature are partially known. This can be achieved by using the product of two matrices: one is the partially known regulation matrix, the other one is the reconstructed activity matrix. If more accurate predictions are demanded, the regulation strengths of TFs and ncRNAs are required, which can be obtained from ChIP-Chip binding significance data [1].

## Conclusion and discussion

The rapid progress of various high-throughput experiment techniques makes more and more biological data available, which makes it possible to quantitatively study regulation mechanisms in a systematic manner. Especially, in recent years, ncRNAs have been revealed to play important regulation roles in many critical pathways. In this paper, we modeled the regulatory system involving two levels (transcription and post-transcription) by a set of closed biochemical reactions. A novel mathematical model is developed to infer regulator activities by considering both transcriptional events and post-transcriptional events and solved by a new iterative algorithm. Experiments on both synthesized data and *E. coli *biological data demonstrated the effectiveness of our method.

A limitation in our current approach is that the reconstructed activities are somewhat dependent on the initial setting of regulation matrices. Although there is also such a problem in other similar studies, they usually use some reduction or other methods to heuristically make the algorithm converge to a unique solution. We will adopt the similar strategy by further incorporating biological constraints [12] in the future research. In addition, with the fact that most of ncRNAs are inhibitors and extremely few are activators (still some), more appropriate model in the future is needed to embody this observation, which should be different from conventional TF-gene regulation models. With the increasing knowledge about the regulation mechanism of ncRNAs, the system model can be modified to be more biologically meaningful. As a future research topic, we will systematically investigate the post-transcriptional effects of ncRNAs in regulation mechanisms of *E. coli *and other organisms.

## Methods

In this work, the regulatory interactions between TFs, ncRNAs and target genes are modeled by a closed biochemical reaction system. With mass action law kinetics and quasi-equilibrium assumption, the concentrations of TFs, mRNAs and ncRNAs and the regulatory relationships between them form a set of log-bilinear equations, which in turn can be transformed into a set of bilinear equations (6). Usually, due to data noise and internal uncertainty, there is generally no exact solution satisfying this set of equations, therefore, we formulate an optimization model to find the solutions with minimum errors between experimental observations and reconstructed data. Due to the nonlinearity of the optimization model, we adopt an iterative strategy to solve it. The optimization model and the algorithm details are as follows.

### Optimization model

Although there is no approximation on the mathematical manipulation except quasi-equilibrium assumption, the model that we formulated above is actually a linear form. Given the expression profiles of target genes, we aim to reconstruct regulator activities and regulation strength so as to make the model most consistent, i.e.

(7) $$\underset{J,M,A,R}{\min}|X-JA+MR|.$$

Usually some prior knowledge on *J *and *M *may be available. For example, ChIP-chip data provides the regulatory relationships between TFs and target genes [34]. The ncRNA-protein interaction database (NPInter) stores ncRNA-protein interaction data covering eight category functional interactions in six model organisms [35]. TF-gene interactions and ncRNA-mRNA interactions can be combined into a two-level regulatory network with common targets as connectors. Such network reflects both transcriptional events and post-transcriptional events. However, the prior knowledge on *J *and *M *is not sufficient because it only provides the binary regulatory relationships without concrete regulation strengths. Thus, the optimization problem formulated above is a nonlinear optimization problem. We will solve this problem by employing partial prior knowledge and an iterative algorithm.

### Iterative algorithm

Since the model (7) is nonlinear, conventional algorithms not only suffer from the computational complexity problem for large scale networks but also are easily trapped into local minima. Here, instead of using conventional optimization techniques, we develop an iterative algorithm efficiently to solve the optimization problem. Although this algorithm cannot guarantee global optimal solutions, in each iteration, two linear programming (LP) models are solved, which is expected to improve the efficiency and accuracy due to polynomial time exact algorithms of linear programming. The steps of such an iteration procedure are described as follows.

• Step 0: Initialize the matrices *J *and *M *using random matrices with entries between -1 and 1 according to the prior knowledge on *J *and *M*. For example, if we already know that TF_*j *_does not regulate the *i*th gene, then *J*_*ij *_= 0. If we know TF_*j *_positively regulates the *i*th gene, then *J*_*ij *_> 0. There are similar operations on *M*.

• Step 1: Given *X*, *J *and *M*, the regulation activity matrices *A *and *R *can be obtained by

(8) $$\underset{A,R}{\min}|X-JA+MR|$$

which is a linear programming problem.

• Step 2: Given *X*, *A *and *R*, the regulation strength matrices *J *and *M *can be obtained by

(9) $$\underset{J,M}{\min}|X-JA+MR|+\lambda (|J|+|M|)$$

with the prior knowledge on *J *and *M *formulated as linear constraints. The optimization problem in this step is also a linear programming.

• Step 3: Repeat Step 1 and Step 2 until convergence condition is met.

In above iterative algorithm, assume the expression matrix *X *= [*x*_*it*_]_*m *× *n*_, *A *= [*a*_*jt*_]_*c *× *n*_, *R *= [*r*_*st*_]_*k *× *n*_, *J *= [*J*_*ij*_]_*m *× *c *_and *M *= [*M*_*is*_]_*m *× *k*_, then the optimization model (8) can be rewritten as

(10) $$\underset{a_{jt},r_{st}}{\min}\sum_{i=1}^{m}\sum_{t=1}^{n}|x_{it}-\sum_{j=1}^{c}J_{ij}a_{jt}+\sum_{s=1}^{k}M_{is}r_{st}|.$$

Let

$$u_{it}+v_{it}=|x_{it}-\sum_{j=1}^{c}J_{ij}a_{jt}+\sum_{s=1}^{k}M_{is}r_{st}|$$

and

$$u_{it}-v_{it}=x_{it}-\sum_{j=1}^{c}J_{ij}a_{jt}+\sum_{s=1}^{k}M_{is}r_{st},$$

where *u*_*it *_≥ 0, *v*_*it *_≥ 0, then the optimization model (8) can be rewritten as a standard linear programming as follows:

(11) $$\begin{array}{ll} \underset{a_{jt},r_{st},u_{it},v_{it}}{\min} & \sum_{i=1}^{m}\sum_{t=1}^{n}\left(u_{it}+v_{it}\right)\\ s.t. & u_{it}-v_{it}=x_{it}-\sum_{j=1}^{c}J_{ij}a_{jt}+\sum_{s=1}^{k}M_{is}r_{st},\\ & u_{it}\geq 0,v_{it}\geq 0, \end{array}$$

where *s.t*. means "subject to". Similarly, the optimization model (9) can be rewritten as

(12) $$\underset{J_{ij},M_{is}}{\min}\sum_{i=1}^{m}\sum_{t=1}^{n}\left|x_{it}-\sum_{j=1}^{c}J_{ij}a_{jt}+\sum_{s=1}^{k}M_{is}r_{st}\right|+\lambda (\sum_{i=1}^{m}\sum_{j=1}^{c}|J_{ij}|+\sum_{i=1}^{m}\sum_{s=1}^{k}\left|M_{is}\right|).$$

Further letting *y*_*ij *_+ *z*_*ij *_= |*J*_*ij*_*|*, *y*_*ij *_- *z*_*ij *_= *J*_*ij*_, and *ω*_*is *_+ *ξ*_*is *_= |*M*_*is*_*|*, *ω*_*is *_- *ξ*_*is *_= *M*_*is*_, then the model (9) becomes a standard linear programming as follows:

(13) $$\begin{array}{ll} \underset{u_{it},v_{it},y_{ij},z_{ij},\omega_{is},\xi_{is}}{\min} & \sum_{i=1}^{m}\sum_{t=1}^{n}\left(u_{it}+v_{it}\right)+\lambda [\sum_{i=1}^{m}\sum_{j=1}^{n}\left(y_{ij}+z_{ij}\right)+\sum_{i=1}^{m}\sum_{s=1}^{k}\left(\omega_{sj}+\xi_{sj}\right)]\\ s.t. & u_{it}-v_{it}=x_{it}-\sum_{j=1}^{c}(y_{ij}-z_{ij})a_{jt}+\sum_{s=1}^{k}(w_{is}-\xi_{is})r_{st},\\ & \omega_{is}-\xi_{is}\geq 0,\\ & u_{it},v_{it},y_{ij},z_{ij}\omega_{is},\xi_{is}\geq 0 \end{array}$$

These standard linear programming problems can be solved efficiently by any LP software such as GLPK linear programming/MIP solver. When the iterative algorithm converges, the obtained matrices *A *and *R *are the solution, i.e. the regulation activities of TFs and ncRNAs.

## Abbreviations

TF: transcription factor; ncRNA: non-coding RNA; miRNA: microRNA; sRNA: small non-coding RNA; RPII: RNA polymerase II; NCA: network component analysis; PCA: principle component analysis; ICA: independent component analysis; NPInter: ncRNA-protein interaction database; LP: linear programming;

## Competing interests

The authors declare that they have no competing interests.

## Authors' contributions

RSW and LC proposed the main idea and designed the research. RSW performed the experiments. GJ prepared the data materials. GJ and XSZ gave valuable suggestions and improvements. LC and XSZ supervised the project. All authors wrote and approved the manuscript.

## Supplementary Material

Additional File 1 **Hypothetical network model**. This file contains the regulator activities and target gene expression profiles used in the hypothetical network model. Click here for file

Additional File 2 **The whole two-level regulatory network**. This file contains all regulatory interactions between TFs, mRNAs and ncRNAs in the two-level regulatory network used in this work. Click here for file
